# Modeling of Burst Impulse Noise Errors in an In-House M-QAM-Based Power Line Communications Channel Using the Fritchman–Markov Model

**DOI:** 10.3390/s23156659

**Published:** 2023-07-25

**Authors:** Akintunde O. Iyiola, Ayokunle D. Familua, Theo G. Swart, Thokozani Shongwe

**Affiliations:** 1 Department of Electrical and Electronic Engineering Science, University of Johannesburg, Auckland Park, P. O. Box 524, Johannesburg 2006, South Africa; familuakunle@gmail.com (A.D.F.); tgswart@uj.ac.za (T.G.S.); 2 Department of Electrical and Electronic Engineering Technology, University of Johannesburg, Doornfontein, P. O. Box 17011, Johannesburg 2028, South Africa; tshongwe@uj.ac.za

**Keywords:** error sequence, Fritchman–Markov model, impulse noise error, power line communication, quadrature amplitude modulation, software-defined radio

## Abstract

Within the power line communication (PLC) network, a large number of electronic devices are connected, and environmental factors can cause unusual behavior, leading to high-amplitude impulse noise in the received signal and, as a result, packet losses and burst errors in the data that are sent. Burst errors make it difficult to send data over power line channels efficiently and accurately. Analyzing error patterns with intelligent techniques can provide valuable insights into data transmission efficiency, enhance transmission quality, and optimize PLC systems. This research proposes a three-state Fritchman–Markov chain-based power line communication error model and develops a software-defined PLC system. The goal is to analyze and model the system’s statistical error process. The PLC system’s fundamental error pattern is deduced from the transmission and reception of data on our software-defined (SD) PLC platform. The system is designed with multi-state quadrature amplitude modulation (M-QAM) data transmission and reception techniques. An error pattern consisting of 50,000 bits is obtained by comparing the bits transmitted with those received using the in-house M-QAM-based PLC transceiver system. The error characteristics of the newly developed M-QAM SD-PLC system are precisely modeled using the error model. Examining the burst error statistics of the reference error sequences of the SD-PLC system and the three-state Fritchman–Markov error model reveals striking similarities. According to the results, the error model accurately represents the error characteristics of the developed M-QAM SD-PLC system. The proposed three-state Fritchman–Markov chain-based error model for PLC has the potential to provide a comprehensive understanding of the error process in PLC. Additionally, it can assess error control strategies with less computational complexity and a shorter simulation time.

## 1. Introduction

Power line communication (PLC) has received considerable attention in recent years as a reasonable alternative for secure, reliable, and low-cost communication technologies for smart grids [1], smart metering [2], smart homes [3], and Internet-of-Things [4] communication applications, as it leverages existing universal electrical power networks. Moreover, PLC provides innovative and highly efficient networking capabilities, without needing additional cables to be connected to the power supply in hostile wireless environments where radio propagation loss is high, such as underground mining environments [5]. However, the PLC network, on the other hand, was not primarily designed for data transmission; hence, the transmission of a communication signal over an electrical power line network can suffer from various degrees of impairment, which are grouped according to an extensive literature study into signal attenuation, multi-path propagation, and non-Gaussian noise, making the communication channel unreliable and hostile [6,7].

The main impairment affecting PLC systems’ reliability is the additive power line noise [7,8,9,10]. This noise is generated by electrical devices connected to power lines, external noise, and interference coupled to the power lines through radiation or conduction [11]. The PLC channel noise can be divided into several categories based on the source, level, and physical characteristics. According to [7], noise in PLC channels can be classified as colored background noise, periodic impulsive noise synchronous with the mains, periodic asynchronous impulsive noise, aperiodic impulse noise, and narrow-band interference from both internal and external sources. Comprehensive information on the sources and effects of these noise types on reliable data transmission on the PLC channel can be found in [10,12]. For modeling flexibility, these five types of noise found in PLC are typically divided into two primary categories: background noise and impulsive noise.

Impulse noise is the most challenging type of noise to deal with in PLC systems, and it is the primary cause of data transmission errors when communicating via an indoor power line channel [12,13,14]. In-house power line communication (PLC) systems can be affected by impulse noise, which can introduce disturbances and interfere with the transmission of data signals over the power line network. Unlike background noise, which is stationary, impulse noise occurs at short intervals but has a high power spectral density (PSD) that is up to 40 dB higher than that of background noise [15]. Impulse noise is a type of non-stationary stochastic electromagnetic interference, with random spikes in signal strength and changing spectral content. Impulse noise in PLCs can originate from various sources, including electrical appliances, switching operations in residential and industrial environments, power fluctuations, and external sources such as lightning or radio frequency interference (RFI), among other factors [10,16,17]. The operation of appliances such as refrigerators, air conditioners, and dimmer switches can generate impulsive disturbances due to their switching actions or motor operations.

Additionally, power line network connections and power supply fluctuations within the household can cause transient impulses that affect the quality and reliability of the PLC signal. Furthermore, external sources of noise, such as nearby lightning strikes [18] or RFI from electronic devices, can couple into the power line network and manifest as impulsive noise, further degrading the performance of in-house PLC systems. Undoubtedly, one of the most important natural sources of impulse noise is lightning (atmospheric discharge). It happens when electricity discharges between clouds or between a cloud and the ground, causing the sudden release of energy that manifests as a bright flash and a loud thunderclap. Comprehending the statistical properties of impulse noise, its effects on data transmission, and methods to mitigate the noise’s negative effects on PLC networks is therefore essential. Further, due to the unique noise characteristics of PLC channels, modulation and coding methods designed specifically for Gaussian channels may not be optimal for PLC channels. For these reasons, there has been a rise in interest in the modeling of and research into the statistical characteristics of PLC noise to assist in developing and designing reliable PLC systems. In this study, we examine the impact of additive impulse noise on the data transmission and functionality of an indoor low-voltage (LV) PLC system.

A review of multiple noise measurements in PLC environments, in particular, shows that noise in PLC networks is not Gaussian, is correlated, and is very impulsive [7,14,19]. The Gaussian distribution, which describes the statistical properties of additive noise in conventional communication systems, is not sufficient to model the impulsive nature of additive impairments on a PLC channel [13]. The Class-A model [20] and the Bernoulli–Gaussian model [21] are commonly employed to characterize impulse noise PLCs. Nevertheless, due to their lack of memory, these mathematical models fail to account for a crucial characteristic of real channels: impulsive burst errors. However, experimental research by Zimmermann et al. [7] has demonstrated that the partitioned Markov model (PMM) provides an accurate statistical model of the noise process in the PLC context.

Fritchman [22] devised the PMM and used it to describe how errors in binary bursty channels depend on statistics. Zimmermann et al. [7] also showed that PMM is an efficient model for accurately simulating additive noise in PLC compared to experimental measurements. This is because PMM can take into account how impulses occur in bursts. Subsequently, the Fritchman–Markov Model (FMM) [22] has been utilized to study the statistical properties of noise and disturbances and also to model noise processes in a wide range of digital communication problems [14,23,24]. In [23], Familua and Cheng used the Fritchman model and the Baum–Welch algorithm to model the noise and disturbances on the in-house CENELEC A-band channel based on experimental measurements. Wang et al. [25] modeled impulsive noise over an automotive power line communication network with an improved Markov model. Familua et al. [24] also developed different phase-shift keying (PSK) modulation schemes using software-defined radio and the universal software radio peripheral (USRP) as an experimental platform for the modeling of noise and disturbances on the low-voltage in-house CENELEC A-band environment with a first-order Markov model. Myint et al. [26] recently investigated the error behaviors in LDPC-coded and Turbo-coded OFDM data transmission systems in a 5G environment. They also used two-state Markov chains to model the transmission error process. However, the QAM-based SD-PLC transceiver system and the real-world investigation of noise processes using the Fritchmam model in the low-voltage indoor CENELEC C-band PLC environment have received relatively little attention in the PLC literature.

Due to the characteristics of power line networks and how susceptible they are to different types of noise, it is important to choose modulation and coding schemes for PLC networks carefully so that they can deal with the effects of hostile channel conditions that are not typical of other well-known communication channels. Single- and multi-carrier modulation schemes are attractive candidates for NB-PLC applications and have been examined in the literature [27] and adopted in practical applications and PLC technology standards [28]. Furthermore, the G.hnem project was introduced by the International Telecommunications Union—Telecommunications Sector (ITU-T) in January 2010 [28]. The ITU-T G.hnem standard incorporates quadrature amplitude modulation (QAM) as the designated orthogonal frequency division multiplexing (OFDM) component within its specification. To this end, this research selected M-QAM modulation techniques developed in software-defined radio PLC platforms to study the noise process and how it affects data transmission in a CENELEC C-band PLC environment.

To mitigate the adverse effects of impulsive noise in PLC applications, it is crucial first to understand its statistical properties and then model its behavior. Accomplishing these goals requires the measurement and modeling of impulse noise errors [29]. To model PLC noise error conditions, assessing the harmful effects of noise and how they influence data transmission is necessary. The transmission error model also allows for the evaluation of system reliability and the contrast and comparison of various modulation and error correction methods that could be used to mitigate channel disturbances. The channel error model also permits the evaluation of the system’s dependability and comparison of the various modulation and error correction techniques that could be implemented to reduce channel disturbances. Choosing accurate and suitable models that depict the PLC noise error characteristics is crucial to improve the implementation of a new PLC evaluation and optimization. Moreover, when implementing error control strategies, it is essential to understand the statistical error distribution patterns, because one must know whether the techniques used have adequate error identification and correction capabilities for error mitigation and reliable data transmission. Consequently, the primary objective of this study is to examine the harmful effects of impulse noise errors on data transmission, and also the selection of a reliable model that depicts the PLC noise error pattern and enables the design of optimal or sub-optimal receivers, as well as overall system optimization in an indoor low-voltage NB-PLC system that operates in the C-band frequency range of the European Committee for Electrotechnical Standardization (CENELEC).

To conduct experimental measurements of error sequences in an in-house PLC environment, we developed an M-QAM-based SD-PLC transceiver system that is subsequently used as a test bed to obtain error sequences. Different countries use distinct PLC standards, and PLC channels have unique challenges. This makes it very difficult to design a universal PLC transceiver [30]. Greater flexibility in the design of PLC transceiver systems is required to overcome these obstacles and achieve the design of a reliable PLC system. Hence, this study employed and developed a software-defined power line communication (SD-PLC) transceiver system with flexibility and programmability to record error sequences and model impulse noise errors in an in-house and narrowband (NB) CENELEC C-band PLC environment. The network comprised many connected electrical devices functioning as impulse noise generators for real-time data transmission and error sequence measurements for different M-QAM schemes. The impulse noise error was measured experimentally in this study using two distinct measurement scenarios. These were scenarios with weakly interrupted and strongly interrupted noise. A single device was connected to the channel in the weakly interrupted case, whereas, in the strongly interrupted case, two indoor electrical devices were used as noise generators for the channel.

We previously presented some preliminary results from this work [30], which focused on the experimental evaluation of the effects of impulsive noise on transmitted data while using a single device as an error generator. Data sent over an indoor SD-PLC were tested under a weakly interrupted scenario in which a compact fluorescent lamp (CFL) bulb and a hairdryer were turned on and off repeatedly. This investigation built upon earlier studies by considering weakly and strongly interrupted noise conditions. Several tests were performed to investigate how the use of electrical devices in the home affects data transmission over power line channels. In the weakly interrupted scenario, only one device was considered: a laptop charger and a computer monitor. In the strongly interrupted scenario, two household devices were combined: a CFL bulb, a hairdryer, a laptop charger, and a computer monitor. A comprehensive investigation was conducted to determine how the uncoordinated turning on and off of different appliances in the home could affect the quality of the digital data sent over an indoor SD-PLC system. For more information on the detailed experiments, the SD-PLC system design approach, and the modeling technique used in this study, the reader is referred to [31].

This study makes three primary contributions in the areas that follow. First, an experimental test bed was set up with a software-defined M-QAM-based NB-PLC transmitter and receiver system for the measurement and modeling of impulsive noise errors in a functional in-house NB-PLC environment operating in the CENELEC C frequency band. This transmitter and receiver system used M-QAM modulation techniques (4-QAM, 8-QAM, and 16-QAM), MATLAB/Simulink software, and USRP hardware for real-time data transmission and reception. Second, we used the SD-PLC experimental test bed to transmit real-time data for various M-QAM schemes. This test bed consisted of networked indoor electrical equipment that acted as impulse noise generators on the network and collected experimental data (error sequences). Lastly, we examined the effectiveness of the Fritchman–Markov model (FMM) and the Baum–Welch algorithm (BWA) for modeling impulsive noise error patterns in an in-house NB-PLC environment.

The rest of this work is presented in the following order. Section 2 describes the M-QAM SD-PLC transceiver system model, Section 3 discusses the Fritchman–Markov model used in this study for discrete channel modeling, and Section 4 describes the experimental setup and measurement procedure. Section 5 presents the measurements, modeling, and model analytical validation results. Finally, Section 6 contains the concluding remarks.

## 2. M-QAM SD-PLC System Model

The M-QAM SD-PLC system is a digital communication platform that is highly adaptable, programmable, and intelligent. It performs complex operations simultaneously so that data processing and transfer over the electrical power network can be done efficiently, reliably, and synchronously. With the help of this platform, binary data produced by the PLC transmission system may be generated and sent utilizing a wide range of intelligent digital communication techniques, digital signal processing (DSP) algorithms, SDR, and digital and analog operations across the network. The use of programmable hardware is more economically advantageous when choosing a design platform for a reliable communication system. The same hardware architecture can be used to build multiple communication system scenarios, and the software architecture is easily changed. Second, a digital environment allows for much more precise control of the software and hardware parameters, such as the generated signal bandwidth, oscillator phase and frequency, and filter response. Lastly, intelligent software and hardware for digital signal processing make it easy to use algorithms for data generation and transmission, synchronization, data compression, encryption, data reception, and error correction. Moreover, these algorithms are essential for modern digital communication systems’ development. As a result, the aforementioned factors influenced the decision to conduct this research using a flexible SDR design approach.

This section describes the process of developing digital transceiver systems with SD-PLC. To achieve the primary goal of this study, these systems employed M-QAM modulation schemes and SD hardware for real-time data transmission and reception over an in-house power line channel. Furthermore, the SD hardware is highly flexible, re-programmable, re-configurable, and can be customized using the universal software-defined radio peripheral (USRP).

**Figure 1:**
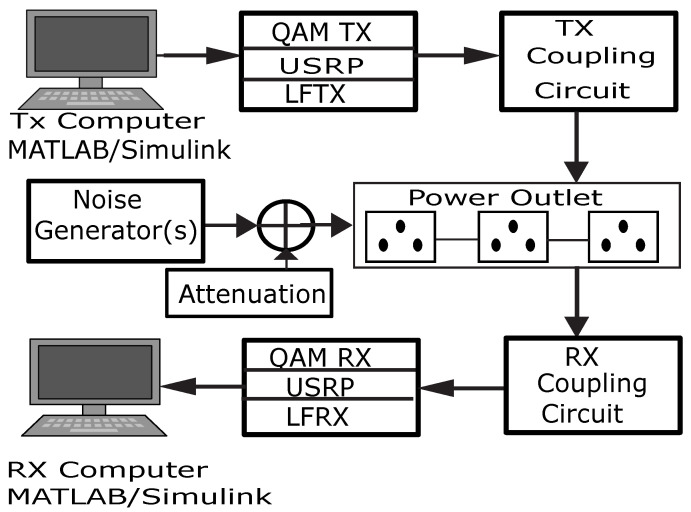
The M-QAM SD-PLC transceiver’s architecture. Adapted from [31].

Figure 1 illustrates the architectural layout of the M-QAM-based SD-PLC transmitter and receiver system utilized in this research.

National Instruments (NI) subsidiary Ettus Research developed and manufactured the SD hardware platform used in this study. A flexible SD transceiver, the universal software-defined radio peripheral (USRP), is used in various communications applications. It is a robust, modular, and utterly programmable SD device. As depicted in Figure 1, the coupling of the USRP transceiver, the PLC coupling circuits, the power outlet serving as the channel, signal processing software, and personal computers (PCs) facilitates the prototyping of PLC systems. The USRP N210 with LFTX transmitter and LFRX receiver daughterboards was connected to an Ethernet network, and the transmit and receive power line coupling interface was used to connect to the PLC channel to facilitate the real-time transmission and reception of data with the MATLAB and Simulink software.

Connecting the transmitter and receiver to the PLC channel required coupling interfaces, which are also crucial components of the PLC system. It acts as a filter, blocking the power signal while letting the communication signal pass through the channel. It also provides galvanic isolation to protect the sensitive parts of the transceiver from the mains voltage [6]. Connecting PLC transceivers directly to the electrical network poses challenges due to the intricate nature of the loads connected to power networks and the variability in the impedance at the connection point. The proper coupling interfaces for the sending and receiving of data must be constructed or selected to send and receive data over an LV indoor SD-PLC channel. This study used the USRP transceiver and an off-the-shelf coupling circuit from STMicroelectronics called the STEVAL-XPLM01CPL to send and receive data over the SD-PLC channel.

**Figure 2:**
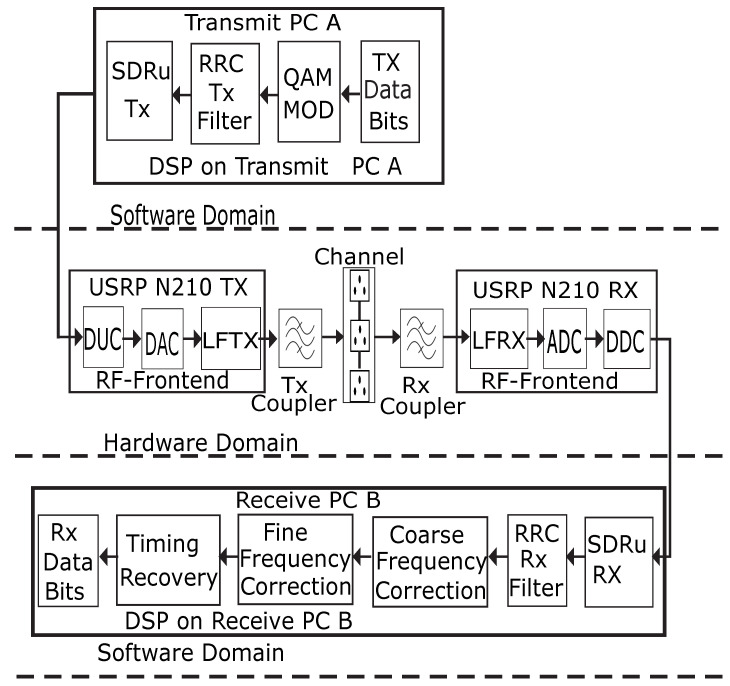
The M-QAM-based SD-PLC transmitter and receiver system. Adapted from [31].

Figure 2 depicts the M-QAM SD-PLC transceiver system. This system comprises the M-QAM transmitter and receiver software, the USRP N210 hardware with the transmit and receive (LFTX and LFRX) daughterboards, and the transmit and receive PLC coupling circuits. It was noted previously that the system performs many complex operations simultaneously to ensure effective data transmission and reception.

The data bits on the host transmit (Tx) PC are first mapped using a QAM modulator to create a modulated baseband signal. The host Tx PC’s modulated signal is then delivered over the gigabit Ethernet data interface to the USRP transmitter through the SDRu transmitter Simulink block. After this, the samples are interpolated, which involves increasing the sampling rate and filtering before digital up-conversion (DUC) [32]. After interpolation, the Tx USRP FPGA executes DUC by transforming the digital complex signal from the baseband to the digital passband equivalent. The digital passband signal generated by the USRP-FPGA is converted into an analog signal by the digital-to-analog converter (DAC). The signal is then routed to the TX daughterboard for further processing. After the TX daughterboard has finished modulating the transmit streams from an intermediate frequency (IF) to the CENELEC C-band operational frequency, the continuous analog output signal is transmitted and superposed on the voltage signal of the power line channel via the transmitter coupling interface.

The system’s receiver component performs multiple tasks when processing the transmitted QAM signal. The reception, synchronization, and demodulation of transmitted signals are a few of these tasks. After the transmit QAM signal has been separated from the power line channel in the first step, the Rx coupling interface sends the signal to the Rx USRP via the RX daughterboard. The RX daughterboard performs filtering and demodulation after sampling analog signals using discrete complex samples from the CENELEC C-band transmit frequency to the intermediate frequency (IF). After sampling the analog signals from the CENELEC C-band transmit frequency to the intermediate frequency (IF) and multiplying them by discrete complex samples, the LFRX daughterboard filters and demodulates them. The resulting digital baseband bit-stream is passed through a digital down-converter (DDC), which filters and decimates the bitstream before reducing the sample rate and eliminating dual-frequency components. The analog-to-digital converter (ADC) changes the analog signal into a digital baseband signal to be further processed. After this, the obtained digital samples are stored in the buffer before being sent through the gigabit Ethernet interface to the host Rx computer for more baseband signal processing. In the final processing phase, the QAM signal demodulator works with the SDRu receiver to demodulate the baseband signals. This allows the QAM signal demodulator to recover the original signal precisely, regardless of whether the channel or noise caused the transmission to be interrupted.

## 3. FMMs for Discrete Channel Modeling

For reliable data transmission over a channel, a typical digital communication system uses a source encoder, a channel encoder, a modulator, a demodulator (both of which are signal processing subsystems), a channel decoder, and a source decoder. In practice, achieving reliable transmission through the channel is invariably hampered by errors caused by destructive variables such as noise and channel distortion. These variables change the relationship between the input and output data sequences, making it more difficult to achieve reliable transmission. Consequently, mathematical channel models must be used to estimate the transmission reliability based on a set of probabilities that relate the output sequences of the channel to the inputs and accurately describe how channel errors affect transmission. This is done to choose a method that effectively protects digital signals from disruptive factors.

**Figure 3:**
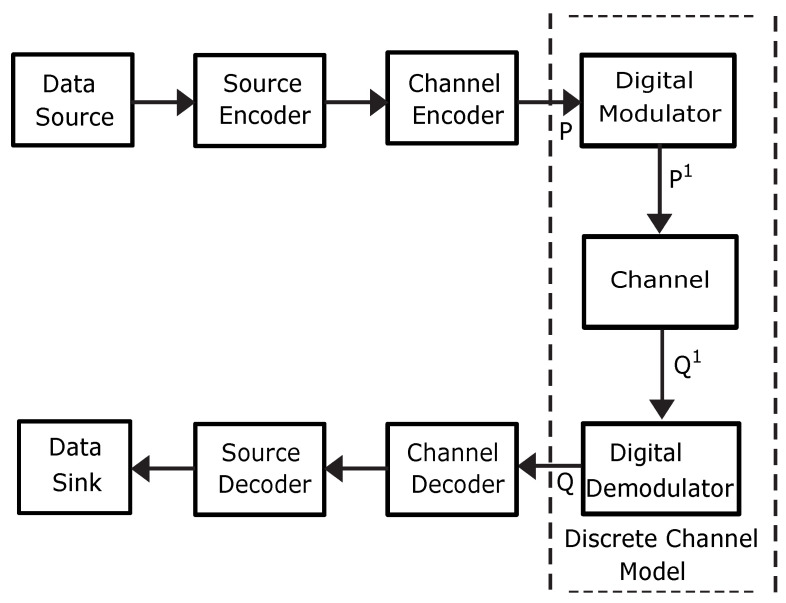
Elements of a discrete communication system [31].

The main components of a discrete communication system [31,33] are shown in Figure 3. All system components located between *P* and *Q* in Figure 3 are referred to as “discrete channels”. Point *P* denotes the transmitter side channel encoder’s output, and point *Q* denotes the receiver side channel decoder’s input. The waveform channel can be found between the two points $P^{1}$ and $Q^{1}$. Moreover, a symbolic sequence *X* defined as $X=\{x_{1},x_{2},\cdots ,x_{k}\}$ is input to point *P*, and a symbolic sequence *Y* defined as $Y=\{y_{1},y_{2},\cdots ,y_{k}\}$ is output from point *Q*, and the two sequences are related. The impairments caused by the physical PLC channel, non-ideal modulator and demodulator, and other devices in the PLC network can be seen as the insertion of an error sequence that exists between the input and output, which is defined as $O=\{o_{1},\ldots ,o_{T}\}$.

Discrete channel modeling (DCM) is a well-established statistical method for the identification and simulation of communication channel impairments and memory activity in digital communication. Similarly, DCM facilitates the development of error correction and coding methods that work well with a real channel. DCM is perfect for modeling digital communication transmission error patterns because digital alphabets are discrete, and a set of probabilities can be used to link the output sequence of the channel to the input sequence to show how channel-induced errors [33,34] affect the transmitted data. These two factors combine to make DCM an ideal candidate for this modeling task.

Discrete communication channels often use a discrete-time Markov sequence to model the mechanisms by which errors are generated. In this model, the model’s state defines the channel’s different states, and a set of transition probabilities represents transitions between states [23,35]. The model’s parameters and structure can be derived from a measured or simulated sequence of errors [33], and multiple symbolic transitions exist between states. DCM first generates a random number before transmitting one symbol to determine the channel state; it then generates another set of numbers for the input-to-output transition.

In the past, DCM approaches were used to analyze and model a wide range of communication channels, such as high-frequency (HF) radio channels [36], indoor PLC channels [23], wireless channels [37], underwater acoustic channels, and, more recently, hybrid PLC-VLC channels [38]. This research combines Markov model concepts with the long burst error characteristics of the transmission medium to gain a deeper understanding of the PLC channel and conduct a thorough analysis of it. In addition, data transfer over the power line channel is more negatively affected by impulse noise than data transmission over other communication channels; therefore, the DCM technique will be effective in describing and characterizing data transmission over power line channels.

### 3.1. The Fritchman–Markov Model

Fritchman [22] used the partitioned Markov chain model to examine the characteristics of the discrete communication channel in 1967. This Fritchman–Markov model (FMM), a finite-state model, statistically describes the channel states and the occurrences of burst errors in a digital communication channel. Fritchman experimented with high-frequency radio channel statistical modeling to demonstrate that Markov chain models can be applied to burst binary communication channels and found that this model can describe actual communication channels.

According to Fritchman’s framework, data transmission can be divided into error-free states (*g*) and error states (N−g). An error state is one in which one or more bits have been received incorrectly, implying a transmission error; in an error-free state, all of its bits have been successfully received without error.

**Figure 4:**
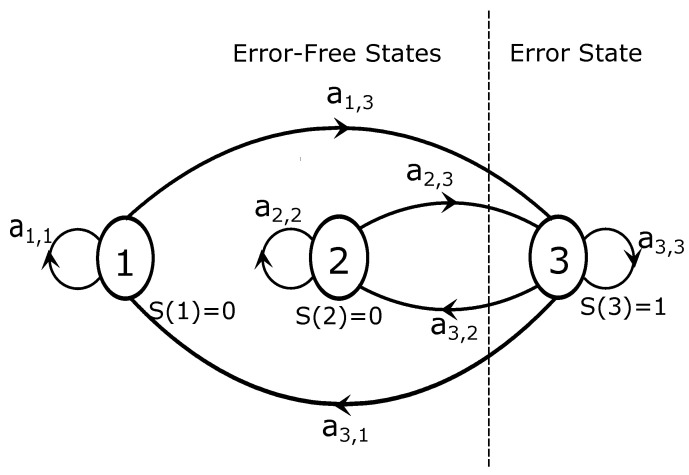
Three-state FMM.

Figure 4 depicts an FMM with three states: two error-free states and one error state. The states S(1) and S(2) are considered error-free as they do not produce any errors, resulting in a representation of zero in the error sequence. In contrast, the state that generates errors, denoted as S(3), is indicated as a value of one in the error sequence due to its error-generating nature. Considering that both cases show error-free transmission, it can be inferred that the two error-free states are identical, as the received bits are accurately received in both instances.

According to Figure 4, it can be observed that the three-state FMM does not permit transitions between states that belong to the same group. This suggests the presence of diverse levels of memory, thus facilitating the modeling of a realistic communication channel model. The choice of two error-free states is based on the fact that the length of the error-free runs in the obtained error sequences is long. A single error-free state would not show this adequately, so the use of two error-free states is justified.

Indoor PLC channels in [24], visible light communication channels in [39], and underwater acoustic channels in [40] were successfully modeled using an FMM with a single-error state. In these works, two or three error-free states with reasonable precision represented the channel burst behavior. As a result, a three-state FMM approach was used in this study.

### 3.2. FMM Parameters

The three-state FMM used for discrete communication channel modeling is defined by three parameters: the N×N state transition matrix *A*, the M×N error probability generation matrix *B*, and the initial state distribution matrix *P*. State transition matrix *A*: For the three-state FMM adopted in this work, the initial state transition probability matrix and the initial values adopted for modeling shall be represented by the following:

(1) $$A=\left[\begin{array}{ccc} a_{1,1} & 0 & a_{1,3}\\ 0 & a_{2,2} & a_{2,3}\\ a_{3,1} & a_{3,2} & a_{3,3} \end{array}\right]=\left[\begin{array}{ccc} 0.8 & 0 & 0.20\\ 0 & 0.70 & 0.30\\ 0.20 & 0.60 & 0.20 \end{array}\right]$$

Although the initial values of the FMM parameters are assumed, it should be noted that the diagonal elements are carefully chosen to depict the error sequence. For the above *A*-matrix, transitions between good states are not possible, which makes the elements $a_{1,2}$ and $a_{2,1}$ zeros. As also seen in Equation (1), the probability of remaining in the two error-free (good) states is high (0.80 and 0.70), depicting the measured bit error sequences, while the probability of remaining in the error (bad) state is lower (0.20).

The error generation matrix *B*: This parameter provides an expression of the probability that a discrete error will take place in the state that has been specified. Taking into account the fact that error-free states are considered to be good states and that errors are always produced in bad states, the error generation matrix for a three-state FMM is as follows:

(2) $$B=\left[\begin{array}{ccc} 1 & 1 & 0\\ 0 & 0 & 1 \end{array}\right]$$

According to Equation (2), the first two columns represent good states, whereas the third represents a bad state. In each state, the probability that there will be no errors is depicted in the first row of the table, while the probability of errors is shown in the second row. Because of this, the model is known as a semi-hidden Markov model because it is only possible to determine which of the good states was responsible for the correctly received bits if there is an error. However, if there is no error, it is not possible to determine which of the good states was responsible for the correctly received bits.

Initial state distribution matrix π: This represents the initial probability of being in any state at any discrete time.

(3) $$\pi =\left[\begin{array}{ccc} 0.45 & 0.45 & 0.10 \end{array}\right]$$

Consequently, the entire set of parameters from Equations (1)–(3) that defines a discrete three-state FMM in compact notation is

(4) $$\begin{array}{c} \Gamma =(A,B,\pi ) \end{array}$$

### 3.3. Estimation of FMM Parameters

After the model and its parameters have been identified and empirical data have been gathered, the essential next step is to evaluate the models’ fit to the experimental data. This evaluation will determine whether or not the model accurately represents the data. The model parameter value that best fits or reflects the experimental data can be used to assess fitness utilizing an approach known as parameter estimation [41]. The maximum likelihood estimate (MLE) and the least-square estimate (LSE) are frequently employed methods in determining the parameter value; this study chose the MLE framework for FMM parameter estimation.

Parameter estimation using the MLE algorithm was chosen due to the algorithm’s favorable properties [41] in these areas: the MLE estimator has sufficient and accurate information about the parameter of interest; the actual parameter value generated by the data can be consistently recovered asymptotically for data with sufficiently large samples; and estimates of the parameter can be obtained asymptotically. When determining model parameter values, the model employs a statistical technique known as the maximum likelihood estimate (MLE). This approach selects values to maximize the probability that the model-represented process produces the observed data.

Advantages such as increased efficiency, improved numerical accuracy, consistency, and invariance of the parameter estimation process have been linked to the use of the MLE method to estimate FMM parameters [41]. Direct numerical maximization (DNM) using a Newton-type minimization algorithm and expectation maximization (EM) [42] using the popular iterative Baum–Welch algorithm (BWA) are two ways to achieve the MLE of FMM parameters.

A reliable method for the assessment of the FMM parameters is the BWA [33,43,44]. One uses an iterative algorithm and dynamic programming techniques to estimate the complete parameter set Γ=(A,B,π) that represents the measured or simulated error sequence [33]. The BWA is an unsupervised machine learning method used to evaluate the FMM’s parameters, corresponding to measured bit error sequences. The algorithm’s training and input data are the experimentally measured error sequences and the initialized FMM parameters. The algorithm modifies the entire set of initialized FMM parameters Γ=(A,B,π) to obtain the most likely parameters representing the measured error sequences. To estimate the model parameters, it uses the maximum likelihood estimation method [42].

Developing the BWA requires the estimation of two different probabilities. The first is the forward-path probability, which is defined as the probability of generating a partial observation sequence in the forward path (starting from the beginning of the data) and arriving at a particular state at a particular frame; it is referred to as the probability of arriving at a particular state. On the other hand, the backward-path probability is the probability of producing a partial observation sequence in the opposite direction from the final data frame, given that the state sequence starts at a particular time.

The BWA can be classified as a specific expectation-maximization (EM) algorithm instance. This algorithm’s various phases include the initial phase, the forward phase, the backward phase, and the update phase. The E-step encompasses both the forward and backward phases of the EM algorithm, whereas the M-step corresponds to the update phase. The forward and backward formulas and computations are employed during the E-step to predict the anticipated hidden states. These predictions are based on the empirical data and the parameter matrices utilized before adjustments. Then, the M-step update formulas are used to improve the parameter matrices so that the observed data and the predicted latent states match up as well as possible.

For a comprehensive and detailed mathematical exposition of the BWA and the modeling technique utilized in this research, please refer to [31,33].

Algorithm 1 demonstrates the step-by-step procedure for the estimation of the FMM parameters using the selected BWA.
**Algorithm 1:** Baum–Welch Algorithm**Input**: Initial model parameter Γ = (A,B,π); training data: the experimentally generated bit error sequence $O=\{o_{1},\ldots ,o_{T}\}$ //( T is the length of the bit error sequence T--length of the bit error sequence. (**Result**: Estimates of *A*-matrix $a_{i,j}$Step 0: Initialize model parameters $A_{0}$, $B_{0}$ and $\pi_{0}$.Step 1: (E-step) forward phase; calculate forward probability parameters.                          //( Three-step calculation of forward probabilities:(
$\alpha_{1}\left(i\right)$ = $\pi_{i}b_{i}\left(o_{1}\right),i=1,2,\cdots ,N.$
//( Initialization process (
$\alpha_{t+1}\left(j\right)$ = $\left[\sum_{i=1}^{N}\alpha_{t}\left(i\right)a_{i,j}\right]b_{j}\left(o_{t+1}\right)$
//( Induction process (for1≤t≤T−1,1≤j≤N.$\mathrm{Pr}[\bar{O}|\Gamma ]$ = $\sum_{i=1}^{N}\alpha_{T}\left(i\right)\beta_{T}\left(i\right)$ //( Termination (Step 2: Calculate the backward probability parameters in the backward phase (E-step). $\beta_{T}\left(1\right)=1,i=1,2,\cdots ,N$    //( Initialization Process ($\beta_{t}\left(i\right)=\sum_{j=1}^{N}\beta_{t+1}\left(j\right)b_{j}\left(o_{t+1}\right)a_{i,j}$, //( Induction Process (for1≤t≤T−1,1≤j≤N.Step 3: Calculate model parameter re-estimation variables and the update process (M-step).
//( Computation of updates at the M-Phase ($\gamma_{t}\left(i\right)=\frac{\alpha_{t}\left(i\right)\beta_{t}\left(i\right)}{\sum_{i=1}^{K}\alpha_{t}\left(i\right)\beta_{t}\left(j\right)},i=1,2,\cdots ,N.$$\xi_{t}\left(i,j\right)=\frac{\alpha_{t}\left(i\right)a_{i,j}b_{j}\left(o_{t+1}\right)\beta_{t+1}\left(j\right)}{\sum_{i=1}^{N}\sum_{j=1}^{N}\alpha_{t}\left(i\right)a_{i,j}b_{j}\left(o_{t+1}\right)\beta_{t+1}\left(j\right)}$//( Determination of the new state transition probability (${\hat{a}}_{i,j}=\frac{\sum_{t=1}^{T-1}\xi_{t}\left(i,j\right)}{\sum_{t=1}^{T-1}\gamma_{t}\left(i\right)},\pi_{i}=\alpha_{1}\left(i\right)\beta_{1}\left(i\right)$,i=1,2,3.Step 4: The algorithm can be stopped if convergence has been reached, and the estimated model parameters can be output. If not, the algorithm will be fed back with the most recent *A* estimates, and steps 1–3 will be repeated until convergence has been reached.

## 4. Experimental Test Bed and Measuring Equipment

In this section, we explain how the SD-PLC transceiver was paired with experimental measurement instruments to create a reliable test bed and the experimental method used to measure impulse noise errors in this study.

Reliable data collection in an experimental study is achieved by using appropriate test equipment and meticulously experimenting. Consequently, after considering the best approach to measuring impulse noise error sequences, a test bed for experiments related to this study was established in the University of Johannesburg’s Auckland Park Communications Laboratory.

**Figure 5:**
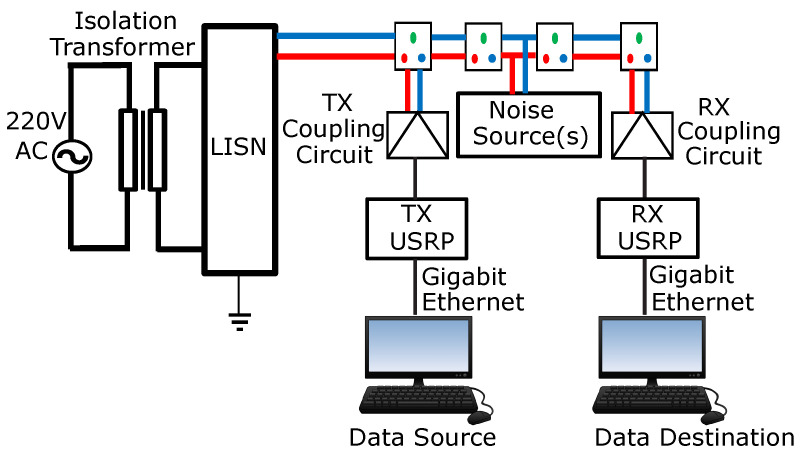
The schematic of the experimental test bed.

**Figure 6:**
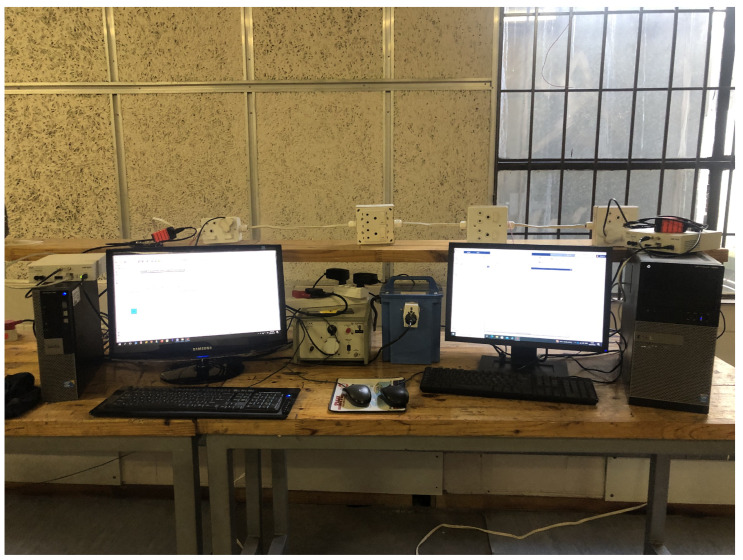
Picture of the experimental test bed.

Figure 5 depicts the architectural layout of the experimental test bed, while Figure 6 depicts a snapshot of the experimental test bed. Collectively, these two figures show the components of the PLC measurement test bed. Components that make up these parts are the isolation transformer, the line impedance stabilizing network (LISN), the power line topology, the transmitting and receiving coupling circuits, the transmitting and receiving USRP, and the transmitting and receiving host PCs.

In power line networks, the impedance fluctuates with the main voltage, making the impedance non-constant. As a direct result, there is an impedance mismatch between the measurement device and the line. To examine the effect of impulsive noise caused by electrically powered home appliances on data transmitted over a power line channel, we set up a test system that isolated the measurement equipment from the mains, removed ground loops, and filtered out electrical noise from the power line.

Using a line impedance stabilization network (LISN) prevents impedance mismatches caused by a fluctuating network impedance, which can compromise the measurement reliability. The LISN is used in conjunction with an isolation transformer for this study. Because the LISN produces large earth leakage currents, it cannot be directly connected to the main circuit, which circuit breakers protect from earth leakage currents.

### 4.1. Network Topology

This section describes the architecture of the power line network (PLN) used for real-time data transfer, measurements, and modeling. This PLN topology served as the testing environment for the SD-PLC system developed in this study. The PLN architecture used in this study is important as the noise parameters vary from country to country due to differences in the mains voltage, power line topology, power line frequency bandwidth, distance, position, and time. The variations in the noise parameters can be attributed to the fact that different countries use different power line configurations, which can result in changes in the frequency bandwidths of power lines.

The CENELEC C-band frequency range is the operating frequency spectrum chosen for the M-QAM SD-PLC transceiver. As the main aim of the power lines was not to transmit high-frequency signals, the PLN thus offers a highly adverse environment for high-frequency signals.

**Figure 7:**
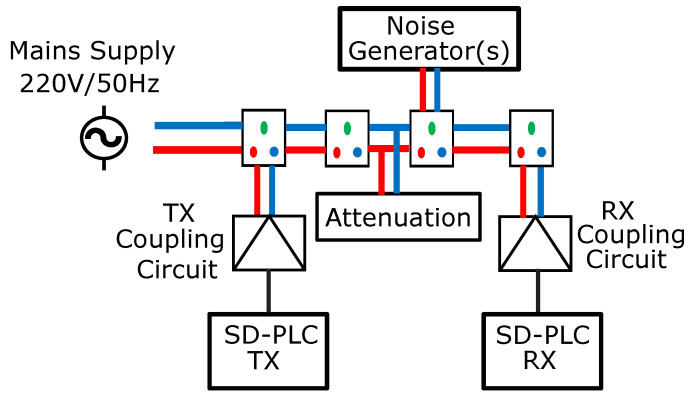
The PLN topology.

Figure 7 depicts the used PLN topology and the test conditions.

### 4.2. Noise Generation Scenarios

An isolation transformer was used to supply electricity from the mains via the LISN to power and manage the loads (laptop charger, computer monitor, CFL bulb, hairdryer, and so on). The load (or loads) was turned on and off at regular intervals to generate impulsive noise while the data transfer was ongoing. The coupling circuit connected the LISN and the load(s) to the power lines. The coupling circuit connected the USRP (transceiver) to the power line.

The transmit and receive USRPs were linked to the test bed through a gigabit Ethernet cable connecting the transmitting and receiving host PCs. The transmit and receive host computers were used to record the noisy bit error sequence that happened when the load on the PLC network was turned on and off. The SD-PLC transceiver test bed in the laboratory can send and receive complex digital baseband M-QAM signals by using an appropriate differential coupling circuit explicitly developed for LV-PLC implementations.

Table 1 presents the fundamental software, hardware, and transmission configuration parameters crucial for this study.

This study examined the impulse noise error sequence by analyzing two distinct scenarios, namely the “weakly interrupted” and “strongly interrupted” scenarios. The weak interruption scenario pertains to the connection of a solitary impulse noise generator to the channel, while the strong interruption scenario involves the connection of two indoor electrical devices that serve as noise generators to the channel. In both situations, the channel experiences interruptions of varying magnitude.

## 5. Experimental Results and Discussion

We conducted comprehensive experiments to determine what happens to digital data sent through an indoor M-QAM-based SD-PLC system [31] when various types of noisy household electrical equipment are turned on and off at random intervals. As mentioned in the previous section, we examined two noise scenarios: one with weakly interrupted noise from a single household device and another with strongly interrupted noise from a combination of two household devices. We used a laptop charger and a computer monitor as noise sources in the weakly interrupted noise scenario. A CFL bulb, a hairdryer, a laptop charger, and a computer monitor were paired to generate noise in the strongly interrupted noise scenario.

This section presents the findings of the statistical error patterns derived from the comparison of transmitted and received bits in the context of this study. Then, using the three-state FMM and BWA techniques, this paper analyzes the impulse noise errors in the SD-PLC channel. Furthermore, the procedure for the validation of the model and analysis of the findings is described in depth.

### 5.1. Error Sequence Measurement Results

Conventionally, a communication system’s bit error rate (BER) performance is used to evaluate its effectiveness. However, the bit error rate (BER) does not provide details about the distribution of channel errors; it does provide important first-order statistical data that are helpful in evaluating channels. In contrast, the bit error pattern provides insights regarding the statistical distribution of errors across a communication channel [36].

The bit error sequence is a sequence of 1’s and 0’s, with 1’s indicating where errors have occurred and 0’s indicating error-free bits. This sequence is created when the received sequence is compared to the transmitted sequence and is distinct from the string of 1’s and 0’s initially transmitted. Next, the M-QAM-based SD-PLC impulse noise error is modeled by applying the three-state FMMs that have been adopted and the BWA to the error sequences that the M-QAM-based SD-PLC has generated. When the error sequence is plotted against the bit number, the statistical distribution of bit errors across the channel can be observed. The scientific community often uses the term “error sequence pattern” to describe this representation. In this experimental investigation, an error sequence consisting of 50,000 bits was obtained by comparing the bits transmitted with those received using the M-QAM-based SD-PLC transceiver system developed in this study.

**Figure 8:**
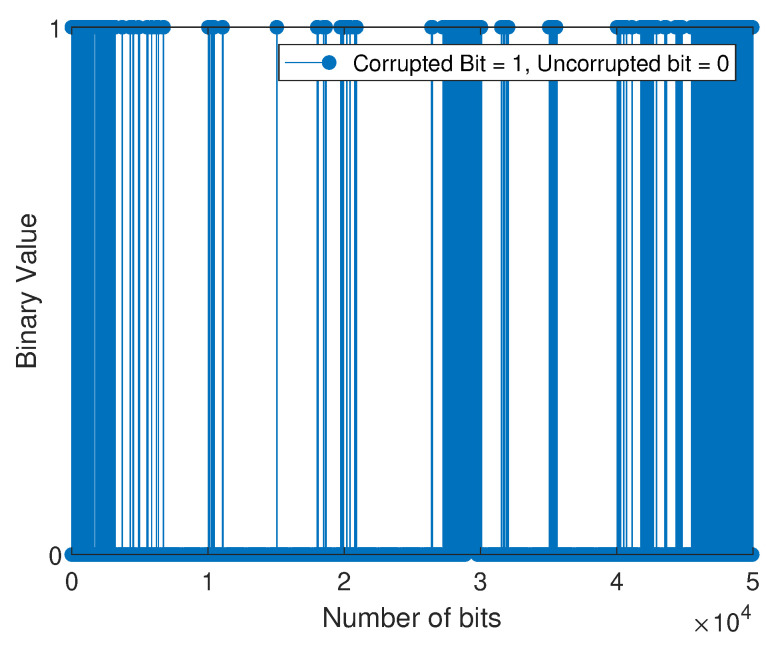
Error sequence for 16-QAM (with laptop charger).

**Figure 9:**
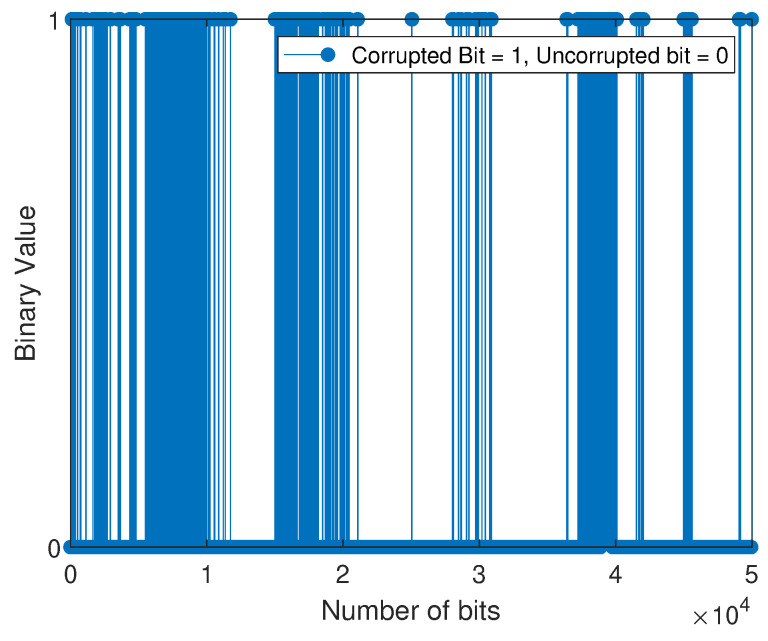
Error sequence for 16-QAM (with a computer monitor).

**Figure 10:**
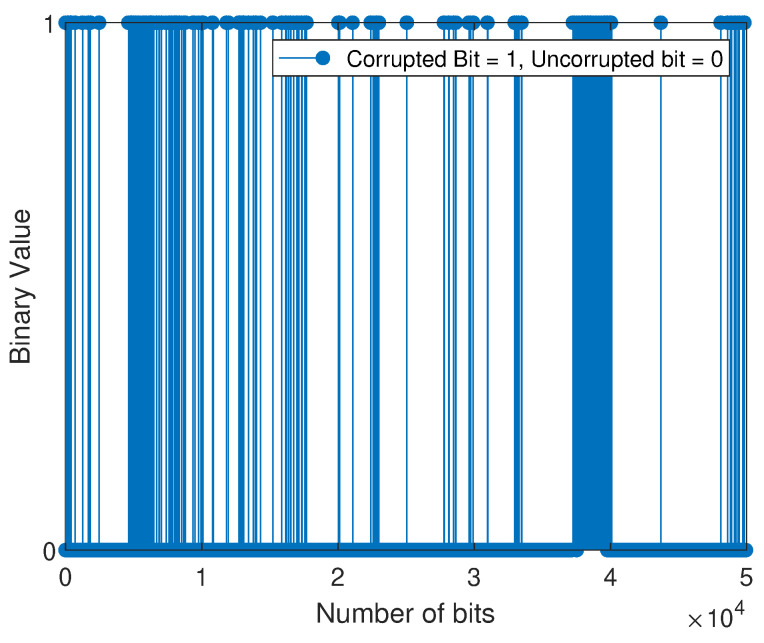
Error sequence for 16-QAM (comprising a laptop charger and a computer monitor).

**Figure 11:**
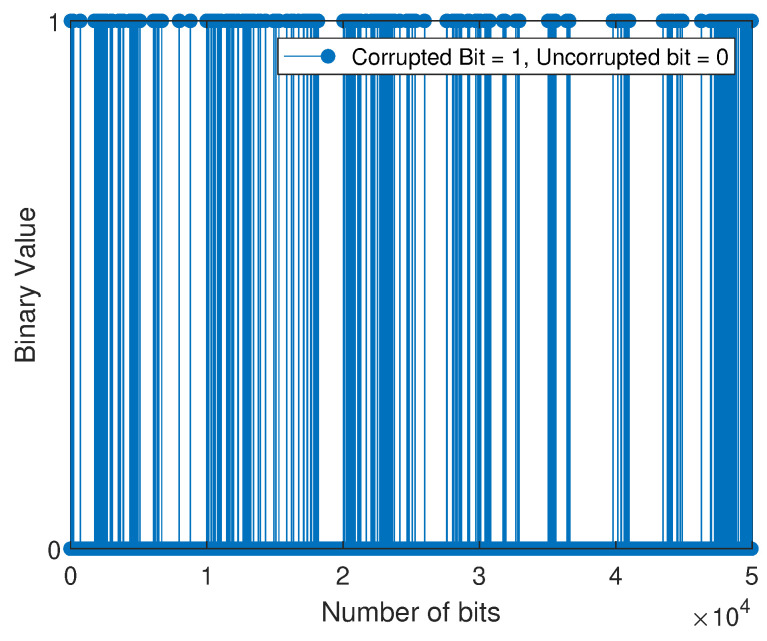
Error sequence for 16-QAM (comprising a CFL bulb and a hairdryer).

For the weakly interrupted noise scenario, Figure 8 and Figure 9 depict the error bit positions of device 1 (laptop charger in a 16-QAM SD-PLC system) and device 2 (computer monitor in a 16-QAM SD-PLC system), respectively. For the case of strongly interrupted noise, Figure 10 and Figure 11 depict the error bit positions of a laptop charger and a computer monitor together in a 16-QAM SD-PLC system, and a CFL-bulb and a hairdryer in a 16-QAM SD-PLC system, respectively.

The study found that impulse noise power affects burst error rates in data transmission. Weakly interrupted noise causes fewer noticeable burst errors, while strongly interrupted noise causes more noticeable ones. Burst errors occur when consecutive bits are corrupted, which can cause synchronization loss and jeopardize data integrity. High-power impulse noise can spread burst errors widely, even across data packets. The investigation also revealed that prolonged burst errors in SD-PLC 16-QAM were significant in both cases and scenarios. Higher-order QAM exhibits a comparatively higher error rate than lower-order QAM and is characterized by reduced efficiency despite its capacity to transmit more data. Using higher-order QAM and experimenting with strongly interrupted noise showed a noticeable rise in the error rate when the impulse noise power was increased. The accurate retrieval of transmitted data was made more difficult due to the heightened impact of the impulse noise, ultimately leading to burst errors in the received data.

Using the developed M-QAM SD-PLC test bed, error sequences of length 50,000 were generated with varying error probabilities and recorded under a variety of experimental measurement conditions. The BWA was trained with the error sequence that was generated by comparing the known binary sequence that was transmitted with the sequence that was received. In addition, the assumed initial values defined in Equations (1)–(3) for the FMM parameters were used as inputs to the BWA.

Following initialization, the BWA was trained using the FMM parameters, with the experimental error sequence functioning as training data.

### 5.2. Estimated State Transition Probabilities

Estimates of transition probabilities describe the model and show which model parameters are most likely to be responsible for the error sequences seen in experiments. These estimates are derived by considering the measured error sequences that have already been provided. Therefore, the estimated state transition probabilities describe how a channel moves from one state to the next.

In Table 2, Table 3, Table 4 and Table 5, the most likely approximate state transition probabilities for Case 1 (laptop charger), Case 2 (computer monitor), Case 3 (laptop charger and computer monitor), and Case 4 (CFL bulb and hairdryer) are shown.

Due to the non-uniform measured error sequence obtained, the high-order QAM system had a higher error probability than the low-order QAM system, which had a lower error probability. Estimates of the probabilities of transitioning between states from Table 2, Table 3, Table 4 and Table 5 depict the non-uniform distribution of the estimated state transition probability. This investigation uses the estimated state transition probabilities to represent the experimental error sequences for each QAM modulation technique, and these probabilities are the exact probability distributions.

### 5.3. Model Validation

Analytical validation of the model is a key stage in determining how accurately the final model captures the channel or event that is the focus of the discussion. This helps to verify that the modeling techniques used are appropriate and that the models realized are the most likely and accurate models that produced the empirically obtained error sequences. Furthermore, it contributes to the validation that the learned models are the most credible and authentic. Analytical validation of the generated FMMs was performed using the log-likelihood ratio, the error-free run distribution, and the error probability.

#### 5.3.1. Log-Likelihood Ratio

The log-likelihood ratio (LLR) is frequently utilized to assess the fitness of a statistical model to a given dataset in statistical modeling. As the term log-likelihood indicates, it is the natural logarithm of likelihood, which means that it is the likelihood expressed as a logarithm. Therefore, it is possible to determine the BWA’s convergence by repeatedly running the algorithm until subsequent estimates of the state transition probabilities show only negligible differences. For a detailed mathematical analysis of the LLR and its representation, please refer to [45].

**Figure 12:**
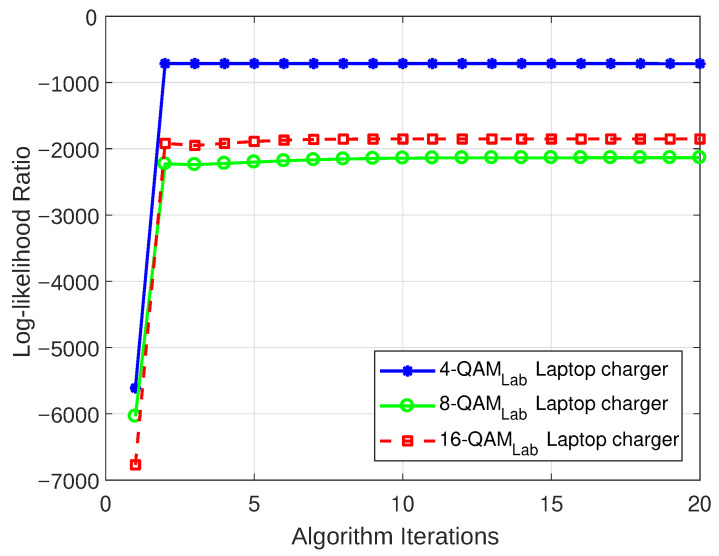
LLR plot for Case 1 (laptop charger).

**Figure 13:**
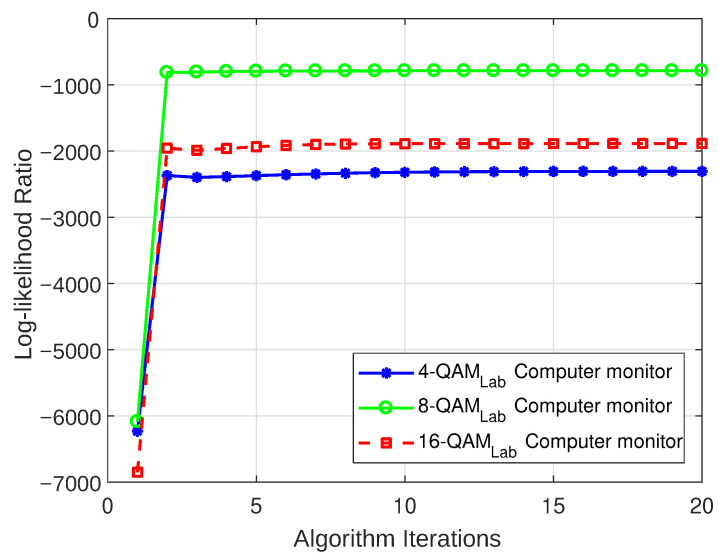
LLR plot for Case 2 (computer monitor).

**Figure 14:**
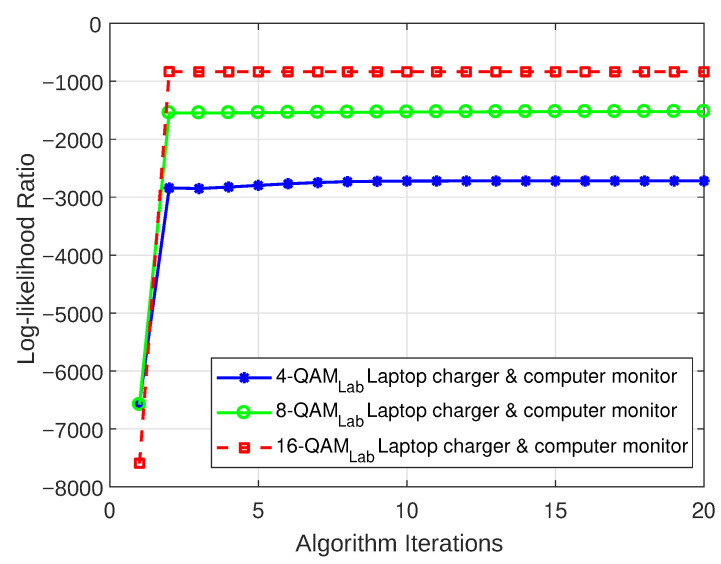
LLR plot for Case 3 (combination of a laptop charger and computer monitor).

**Figure 15:**
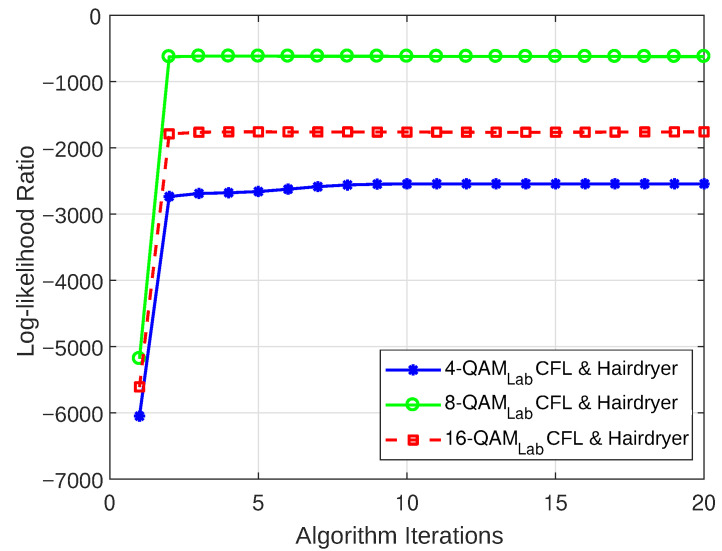
LLR plot for Case 4 (combination of CFL bulb and hairdryer).

The LLR plots for the models that were realized for both the weakly interrupted and strongly interrupted scenarios are depicted in Figure 12, Figure 13, Figure 14, and Figure 15, respectively. These figures show that the models begin to converge at the second iteration with a negative LLR value close to zero, confirming the model’s accuracy.

#### 5.3.2. Probability of Error

Another validation criterion that is used to determine the precision of the FMMs and to check the accuracy of the estimated models is the probability of error. Table 6 shows the error probabilities for both the measured error sequences and the model-generated error sequences for each of the four scenarios that were taken into account for this study.

A closer look at the error probabilities for the measured error sequences in Table 6 reveals that 16-QAM systems are noisier than 8-QAM systems and 8-QAM systems are noisier than 4-QAM systems. This is because, as the constellation size increases (from 4-QAM to 8-QAM or higher), the symbols are forced closer together and thus more susceptible to errors caused by noise and channel impairments. Furthermore, Table 6 shows the relative consistency between $P_{o}$ for experimentally measured error sequences and $P_{m}$ for FMM-generated error sequences for the four considered cases. This validates the accuracy of the estimated or derived models. However, there was a small difference in the model-generated probabilities ($P_{m}$) for 16-QAM and 8-QAM in Case 2, with the model-generated probabilities ($P_{m}$) for 8-QAM being higher than for 16-QAM. The statistical nature of the model-generated error sequence could explain this disparity.

#### 5.3.3. The Error-Free Run Distribution Graph

The probability of transitioning to *m*-consecutive error-free transmissions after an error transmission is depicted by the error-free run distribution (EFRD) graph. The distribution of error-free runs gives a rough idea of how likely a burst or cluster of bit errors is.

**Figure 16:**
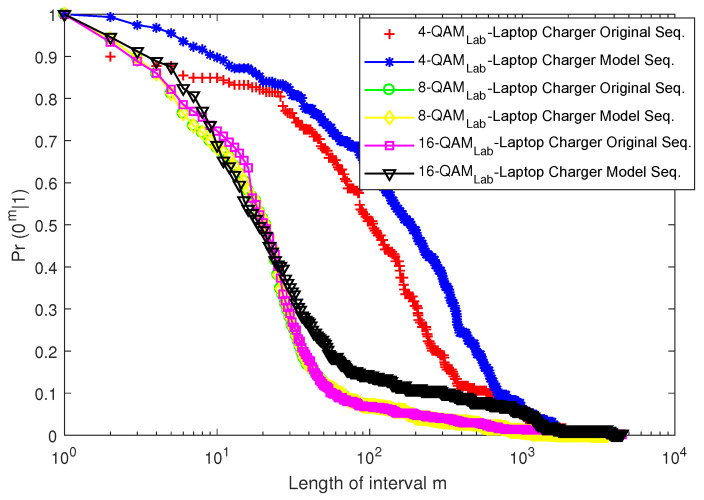
EFRD graph for Case 1 (laptop charger).

**Figure 17:**
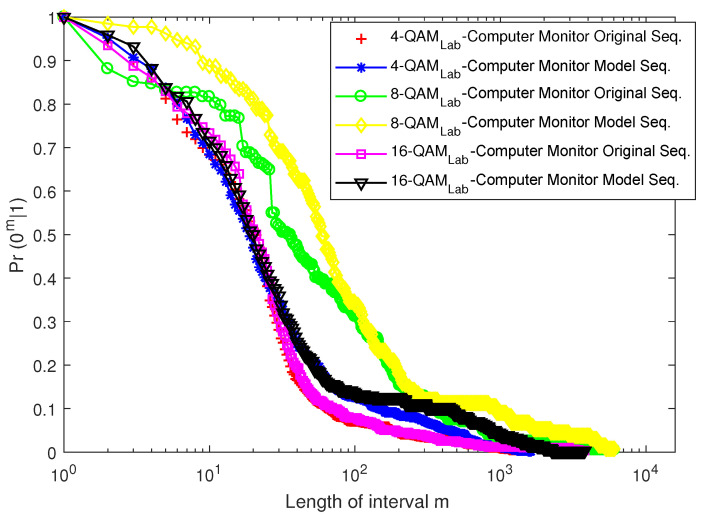
EFRD graph for Case 2 (computer monitor).

**Figure 18:**
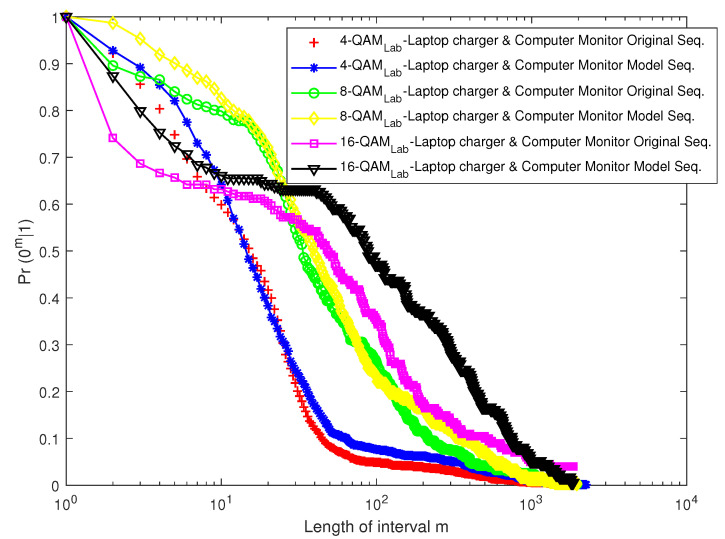
EFRD graph for Case 3 (combination of a laptop charger and computer monitor).

**Figure 19:**
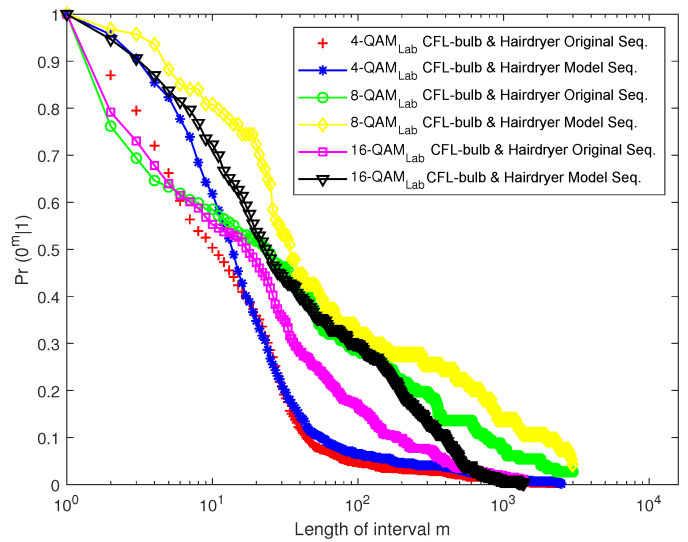
EFRD graph for Case 4 (combination of CFL bulb and hairdryer).

Figure 16 and Figure 17 depict the EFRD plots for Scenario 1 (Cases 1 and 2), whereas Figure 18 and Figure 19 depict the EFRD plots for Scenario 2 (Cases 3 and 4). Calculations were performed to obtain the EFRD probabilities for the measured error sequences. Next, using the estimated model parameters, an error sequence of the same length as the measured error sequences obtained from the experimental measurements was generated. After this, the EFRD was calculated for the measured error sequence and the model error sequence that matched it. The EFRD derived from the experimental measurement was then compared to the EFRD model outputs to ensure that the derived models were correct.

Due to the lower probability of error in Scenario 1 (Case 1), shorter or fewer bursts can be observed. As a result, longer error-free states are present in the error sequence, hence the high values of *m* in Figure 16 and Figure 17. Figure 18 and Figure 19, on the other hand, reveal a longer burst due to a higher probability of error.

For Scenario 1 (Figure 16 and Figure 17), the EFRDs of the measured and model-generated sequences show similar plots, while Scenario 2 (Figure 18 and Figure 19) shows slight differences. The probabilistic nature of the model accounts for the variations, with high variations observed at lower values of *m*. However, the similarities in the EFRDs and the closeness of the probabilities of errors between the measured and respective models confirm the suitability of the FMM.

## 6. Conclusions

This study built a single-carrier M-QAM-based SD-PLC system used as a test bed to determine the statistical properties of data transmission errors and obtain error sequences in an indoor PLC environment. The three-state Fritchman–Markov model and the BWA were used to model the error sequences that were found. Analyses were performed to show that the models were correct based on error sequences that were recorded experimentally at the output of a software-defined M-QAM PLC transceiver. This validation was performed for each modulation scheme studied under different loading conditions. Analytical validation metrics for the realized FMMs include log-likelihood ratios, error-free run distribution graphs, and error probability.

Moreover, the proposed three-state Fritchman–Markov error model was used to study and model how different connected indoor electrical devices affect data transmission on M-QAM-based SD-PLCs working in a noisy environment. The performance evaluation of the M-QAM-based SD-PLC systems for this study showed that the 4-QAM system was the most reliable and the 16-QAM system was the least reliable. As more devices are added to a power line network, the probability of errors and bursts of errors rises. In this study, M-QAM-based SD-PLC system development and experimental channel error assessment led to results that were similar to those in real-world PLN channel situations.

The statistical behavior of the PLC channel impulse noise error sequence caused by electrical appliances connected together in the network was successfully modeled using a three-state FMM with one error state, and the results were satisfactory. Because the experimental error sequences resemble FMM error sequences, FMMs can be used to simulate impulse noise errors in the NB-PLC channel. The models created can also be used to optimize modulation schemes, reduce or eliminate the effects of impulsive noise, develop error correction methods, and improve the overall efficiency of the PLC system.

Further work will be carried out in the next phase, where we will focus on enhancing the discrete channel modeling techniques and their applications to facilitate the design of forward error correction techniques to mitigate PLC channel impairments.

## Figures and Tables

**Table 1:** Hardware, software, and transmission parameters.

Items	Values and Configurations
USRP hardware (TX and RX)	USRP N210
TX daughterboard model	LFTX, (0–30 MHz)
RX daughterboard model	LFRX, (0–50 MHz)
TX computer/USRP TX IP	192.168.20.1 and 192.168.20.2
RX computer/USRP RX IP	192.168.40.1 and 192.168.40.2
Constellation order	4, 8, 16 QAM
Transmitter and receiver gain	Non-tunable (default)
USRP FPGA and firmware rev	003.005.003
CENELEC C frequency	130 kHz
Sample time	6 μs
Sample frequency	200 kHz
TX and RX bit length	50,000
Host PC TX and RX OS	Windows 10 Pro, 64 bits.
Host PC TX and RX Processor	Intel Core i5-4300u.
Host-based software version	MATLAB R2017a (9.2.0.538062)

**Table 2:** Probabilities of state transitions (estimated) in Case 1: laptop charger.

*A*	4-QAM	8-QAM	16-QAM
$a_{1,1}$	0.9988	0.9993	0.9992
$a_{1,3}$	0.0012	0.0007	0.0008
$a_{2,2}$	0.9578	0.9738	0.9529
$a_{2,3}$	0.0422	0.0262	0.0471
$a_{3,1}$	0.0694	0.0145	0.0089
$a_{3,2}$	0.3383	0.0547	0.0905
$a_{3,3}$	0.5923	0.9308	0.9005

**Table 3:** Probabilities of state transitions (estimated) in Case 2: computer monitor.

*A*	4-QAM	8-QAM	16-QAM
$a_{1,1}$	0.9986	0.9994	0.9981
$a_{1,3}$	0.0014	0.0006	0.0019
$a_{2,2}$	0.9386	0.9437	0.9434
$a_{2,3}$	0.0614	0.0563	0.0566
$a_{3,1}$	0.0304	0.0049	0.0269
$a_{3,2}$	0.5864	0.0154	0.0856
$a_{3,3}$	0.3832	0.9797	0.8875

**Table 4:** Probabilities of state transitions (estimated) in Case 3: pairing a laptop charger with a computer monitor.

*A*	4-QAM	8-QAM	16-QAM
$a_{1,1}$	0.9977	0.9968	0.9972
$a_{1,3}$	0.0023	0.0032	0.0028
$a_{2,2}$	0.9400	0.9756	0.6240
$a_{2,3}$	0.0590	0.0244	0.3760
$a_{3,1}$	0.0318	0.0442	0.0237
$a_{3,2}$	0.3252	0.1040	0.0126
$a_{3,3}$	0.6430	0.8518	0.9637

**Table 5:** Probabilities of state transitions (estimated) in Case 4: pairing a CFL bulb with a hairdryer.

*A*	4-QAM	8-QAM	16-QAM
$a_{1,1}$	0.9983	0.9991	0.9969
$a_{1,3}$	0.0017	0.0009	0.0031
$a_{2,2}$	0.9369	0.9666	0.9481
$a_{2,3}$	0.0631	0.0334	0.0519
$a_{3,1}$	0.0219	0.0090	0.0300
$a_{3,2}$	0.2994	0.0221	0.0658
$a_{3,3}$	0.6787	0.9690	0.9042

**Table 6:** Error probabilities for measured original error sequence ($P_{o}$) and model-generated error sequence ($P_{m}$) for Cases 1 (laptop charger), 2 (computer monitor), 3 (combination of a laptop charger and computer monitor), and 4 (combination of CFL bulb and hairdryer).

	4-QAM	8-QAM	16-QAM
Case 1, $P_{o}$	0.0166	0.0338	0.0515
Case 1, $P_{m}$	0.0144	0.0362	0.0536
Case 2, $P_{o}$	0.0323	0.0526	0.0597
Case 2, $P_{m}$	0.0338	0.0568	0.0552
Case 3, $P_{o}$	0.0166	0.0338	0.0515
Case 3, $P_{m}$	0.0144	0.0362	0.0536
Case 4, $P_{o}$	0.0508	0.0627	0.0756
Case 4, $P_{m}$	0.0473	0.0655	0.0758

## Data Availability

Not applicable.

## Abbreviations

The following abbreviations are used in this manuscript:

ADC	Analog-to-Digital Converter
BWA	Baum–Welch Algorithm
CFL	Compact Fluorescent Light
DAC	Digital-to-Analog Converter
DDC	Digital Down-Converter
DCM	Discrete Channel Model
DUC	Digital Up-Converter
FMM	Fritchman–Markov Model
LSE	Least-Square Estimate
LV	Low Voltage
MLE	Maximum Likelihood Estimate
M-QAM	Multi-State Quadrature Amplitude Modulation
NB	Narrow Band
PLC	Power Line Communication
PMM	Partitioned Markov Model
RFI	Radio Frequency Interference
PSD	Power Spectral Density
SDR	Software-Defined Radio
USRP	Universal Software-Defined Radio Peripheral
